# Revisiting the role of interleukin-8 in chronic lymphocytic leukemia

**DOI:** 10.1038/s41598-017-15953-x

**Published:** 2017-11-16

**Authors:** Denise Risnik, Enrique Podaza, María B. Almejún, Ana Colado, Esteban E. Elías, Raimundo F. Bezares, Horacio Fernández-Grecco, Santiago Cranco, Julio C. Sánchez-Ávalos, Mercedes Borge, Romina Gamberale, Mirta Giordano

**Affiliations:** 1 Laboratorio de Inmunología Oncológica, Instituto de Medicina Experimental (IMEX)-CONICET-Academia Nacional de Medicina, Buenos Aires, Argentina; 2 Departamento de Fisiología, Biología Molecular y Celular, Facultad de Ciencias Exactas y Naturales, Universidad de Buenos Aires, Buenos Aires, Argentina; 3 Hospital General de Agudos Dr. Teodoro Álvarez, Buenos Aires, Argentina; 4 Sanatorio Municipal Dr. Julio Méndez, Buenos Aires, Argentina; 5 Instituto Alexander Fleming, Buenos Aires, Argentina; 6 Departamento de Microbiología, Parasitología e Inmunología, Facultad de Medicina, Universidad de Buenos Aires, Buenos Aires, Argentina

## Abstract

The proliferation and survival of malignant B cells in chronic lymphocytic leukemia (CLL) depend on signals from the microenvironment in lymphoid tissues. Among a plethora of soluble factors, IL-8 has been considered one of the most relevant to support CLL B cell progression in an autocrine fashion, even though the expression of IL-8 receptors, CXCR1 and CXCR2, on leukemic B cells has not been reported. Here we show that circulating CLL B cells neither express CXCR1 or CXCR2 nor they respond to exogenous IL-8 when cultured *in vitro* alone or in the presence of monocytes/nurse-like cells. By intracellular staining and ELISA we show that highly purified CLL B cells do not produce IL-8 spontaneously or upon activation through the B cell receptor. By contrast, we found that a minor proportion (<0.5%) of contaminating monocytes in enriched suspensions of leukemic cells might be the actual source of IL-8 due to their strong capacity to release this cytokine. Altogether our results indicate that CLL B cells are not able to secrete or respond to IL-8 and highlight the importance of methodological details in *in vitro* experiments.

## Introduction

Chronic lymphocytic leukemia (CLL) is characterized by the progressive accumulation of clonal CD5^+^ B cells in lymphoid tissues and peripheral blood^1,2^. While the great majority of circulating CLL cells is arrested in G0/G1 phase of the cell cycle, clonal proliferation occurs in lymphoid tissues, where CLL cells receive prosurvival and activating signals from a protective microenvironment^3^. The bidirectional cross-talk between CLL and non-malignant bystander cells is mediated through soluble factors and cell-to-cell contact. Interleukin-8 (IL-8/CXCL8) is one of the first molecules reported to play a key role in CLL biology^4^. Its serum levels are increased in most patients compared to age-matched healthy donors^5,6^ and it is widely accepted that IL-8 prolongs CLL cell survival in an autocrine fashion^5,7–9^. However, there is no reported evidence of the expression of IL-8 receptors, CXCR1 and CXCR2, on circulating CLL cells. Of note, an early report from Ghobrial *et al*.^10^ aimed to determine chemokine receptors expressed by CLL cells found that CXCR1 and CXCR2 were absent in 45 peripheral blood samples, and more recently Levidou *et al*. were neither able to detect CXCR2 on lymph node samples from CLL patients^11^.

In addition, most papers describing the ability of CLL cells to release IL-8 disregard the fact that contaminating monocytes in CLL B cell samples may be its actual source due to their strong capacity to produce IL-8^12,13^. Indeed, very low numbers of monocytes (5–8 × 10^3^/ml) that usually remain in CLL suspensions after standard isolation procedures are able to secrete significant amounts of IL-8 and other proinflammatory cytokines upon stimulation^14,15^. Thus, without a thorough depletion of monocytes from CLL samples, researchers might inadvertently obtain false-positive results regarding the ability of leukemic B cells to release IL-8.

The purpose of the present study was to re-examine the role of IL-8 in CLL and highlight the relevance of methodological procedures when a mixture of cell types is evaluated. To this aim, we conducted experiments to determine if leukemic B cells from CLL patients with different staging and prognosis express CXCR1 and CXCR2 and can be protected from apoptosis by IL-8. As a second important point, we assessed the capacity of purified CLL cells to release IL-8 spontaneously or upon activation.

## Results and Discussion

### Leukemic B cells from CLL patients do not express IL-8 receptors

**Figure 1:**
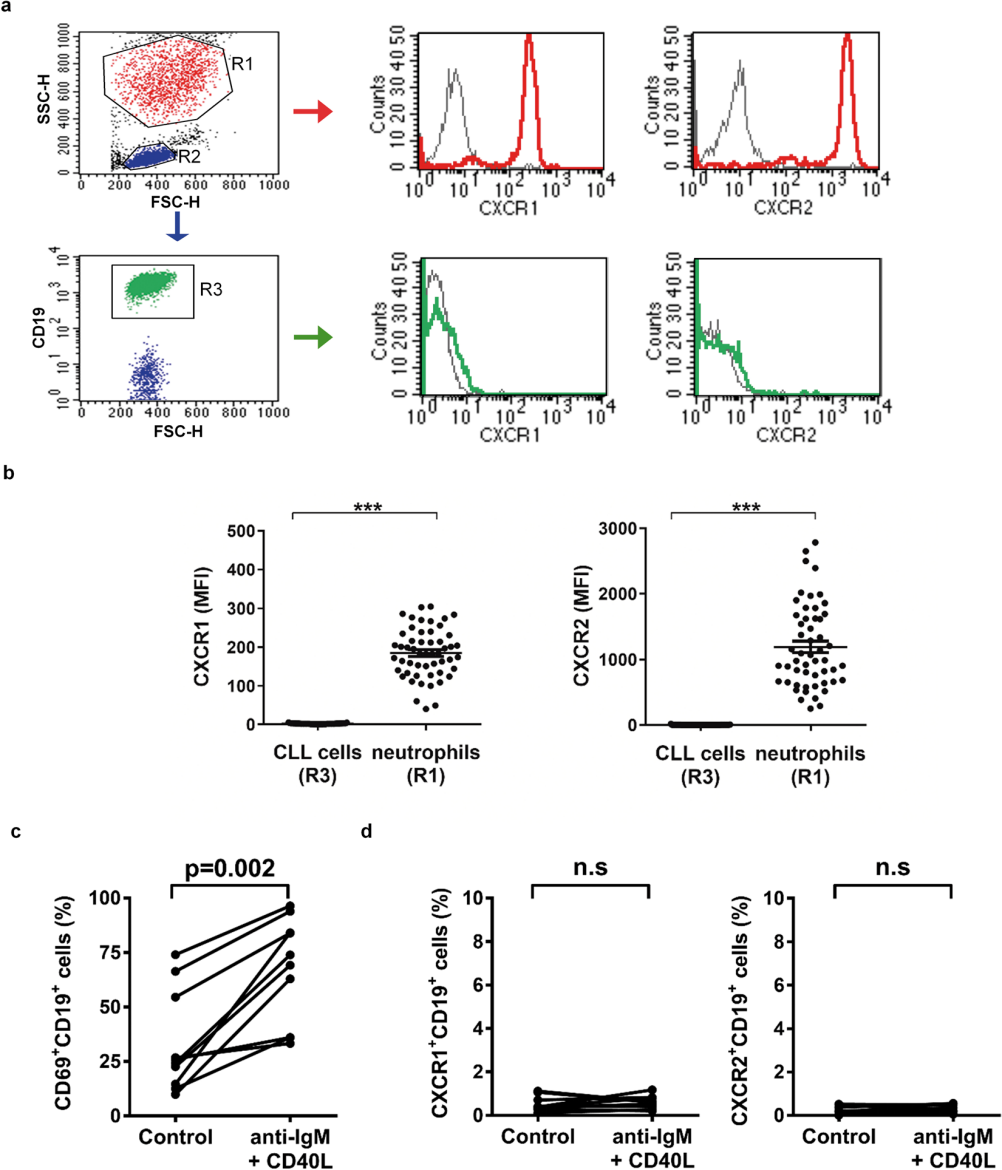
CLL cells do not express IL-8 receptors CXCR1 or CXCR2. Neutrophils (R1) and PBMC (R2) were first discriminated by size (FSC-H) and internal complexity (SSC-H). Leukemic cells were further identified by CD19 expression (R3). (**a**) CXCR1 and CXCR2 expression in CLL cells and neutrophils evaluated by flow cytometry. Shown are representative histograms from one sample with isotype control for each IL-8 receptor in grey line. (**b**) Graphs show mean fluorescence intensity (MFI) of CXCR1 and CXCR2 in neutrophils and CLL cells (mean ± SEM n = 56). Statistical analysis was performed using Mann-Whitney test. Asterisks indicate statistically significant differences (***p < 0.001). Activation of CLL cells does not induce the expression of IL-8 receptors. PBMC (3 × 10^6^/ml) were incubated with immobilized anti-IgM plus CD40L (40 ng/ml) or medium alone (control) for 24 h. Shown are the percentages of CD69^+^ (**c**), CXCR1^+^ and CXCR2^+^ (**d**) leukemic B cells under control or activated conditions (n = 10). Statistical analysis was performed using Wilcoxon test. *p < 0.05, ns: not significant).

Because binding to surface receptors is mandatory for IL-8 to activate cell signaling, we first evaluated the membrane expression of CXCR1 and CXCR2 by flow cytometry in circulating CD19^+^ cells from 56 CLL samples. Clinical and laboratory data of the patients who took part in the present study are depicted in Table 1. Analysis was performed in freshly isolated cell samples or after thawing cryopreserved peripheral blood mononuclear cells (PBMC). Viability was always >90%. As an internal positive control for both IL-8 receptors, neutrophils were added to PBMC suspensions immediately before staining. Representative dot plots and histograms are depicted in Fig. 1a. We found no expression of CXCR1 or CXCR2 in any of the CLL cell samples evaluated (Fig. 1b). Since isolation procedures can temporarily down-regulate the expression of chemokine receptors^16^, we repeated CXCR1 and CXCR2 staining in freshly obtained whole blood from 10 CLL patients. Results confirmed strong expression of IL-8 receptors in neutrophils while CD19^+^ cells were completely negative (Supplementary Fig. S1).

**Table 1:** Clinical and biological features of CLL patients enrolled in the study.

CLL patient #	Gender	Age (years)	Binet	Lymphocytes (×10^9^/L)	CD19^+^ %*	CD38^+^ %^Δ^	CD49d^+^ %^λ^	IGHV mutational status	Experiments performed
**1**	Male	61	C	30.4	82	79	83	n.d.	CXCR1/2; Survival
**2**	Female	81	A	56.7	93	6	7	M	CXCR1/2; IL8 production^I^
**3**	Male	84	A	29.4	81	2	6	M	CXCR1/2; IL8 production^I^
**4**	Male	74	B	129	97	3	40	n.d.	CXCR1/2
**5**	Female	69	A	14.1	86	1	9	M	CXCR1/2
**6**	Male	65	B	67.5	96	37	4	U	CXCR1/2; IL8 production^I^
**7**	Female	65	C	243	97	86	56	M	CXCR1/2
**8**	Female	70	B	90.9	97	0.5	2	M	CXCR1/2; Survival
**9**	Female	75	C	179	97	0.2	99	M	CXCR1/2; IL8 production^I^
**10**	Male	69	C	61.1	93	91	5	M	CXCR1/2; Survival
**11**	Male	84	B	31.2	95	6	1	M	CXCR1/2; IL8 production^I E^; Survival
**12**	Male	82	A	25.5	95	0.6	2	M	CXCR1/2; IL8 production^I^
**13**	Male	60	B	6.90	85	2	31	M	CXCR1/2
**14**	Female	72	C	65.0	87	16	0.4	n.d.	CXCR1/2
**15**	Female	64	A	16.9	69	2	8	n.d.	CXCR1/2
**16**	Male	61	C	58.0	84	0.1	1	M	CXCR1/2
**17**	Male	76	A	24.8	57	8	1	M	CXCR1/2; IL8 production^I E^; Survival
**18**	Male	80	B	104	95	98	26	n.d.	CXCR1/2; Survival
**19**	Female	70	A	45.0	88	1	1	n.d.	CXCR1/2
**20**	Male	71	A	27.7	89	0.3	2	n.d.	CXCR1/2
**21**	Male	72	A	54.0	88	0.8	3	M	CXCR1/2
**22**	Male	64	A	12.3	75	1	n.d	M	CXCR1/2; IL8 production^I E^
**23**	Female	82	A	11.1	78	39	40	n.d.	CXCR1/2
**24**	Male	62	C	2.10	23	3	22	U	CXCR1/2
**25**	Male	70	A	57.0	90	20	n.d	n.d.	CXCR1/2; IL8 production^I E^
**26**	Male	69	B	30.0	71	9	n.d	M	CXCR1/2; IL8 production^E^
**27**	Female	45	C	57.2	94	67	4	U	CXCR1/2; IL8 production^I^
**28**	Male	57	B	20.5	85	0.1	0.2	n.d.	CXCR1/2
**29**	Male	85	B	38.3	91	0.5	n.d	n.d.	CXCR1/2; IL8 production^I E^
**30**	Male	71	B	535	98	15	95	U	CXCR1/2; IL8 production ^E^
**31**	Male	67	B	60.2	90	93	99	U	CXCR1/2; IL8 production^I E^
**32**	Male	68	B	62.3	94	16	0.6	U	CXCR1/2; IL8 production^I E^
**33**	Male	73	B	110	85	44	6	M	CXCR1/2; IL8 production^I^
**34**	Female	87	C	26.8	88	3	99	U	CXCR1/2; IL8 production^I E^
**35**	Male	82	B	5.80	67	5	93	U	CXCR1/2
**36**	Male	61	C	345	97	97	99	U	CXCR1/2; IL8 production^I E^; Survival
**37**	Female	77	B	32.4	91	1	18	M	CXCR1/2; IL8 production^I E^
**38**	Female	72	A	4.00	85	3	9	M	CXCR1/2
**39**	Female	63	A	33.0	87	0.1	20	M	CXCR1/2; IL8 production^I E^; Survival
**40**	Female	58	A	9.94	84	5	5	n.d.	CXCR1/2
**41**	Male	61	B	9.70	62	58	62	U	CXCR1/2
**42**	Female	93	C	24.0	90	0.1	0.1	n.d.	CXCR1/2
**43**	Female	74	B	91.0	95	5	2	n.d.	CXCR1/2
**44**	Male	72	A	33.1	83	11	96	U	CXCR1/2
**45**	Female	76	A	4.40	36	63	50	n.d.	CXCR1/2
**46**	Female	54	A	40.5	94	1	1	M	CXCR1/2
**47**	Female	77	C	24.2	98	0.5	60	n.d.	CXCR1/2
**48**	Male	75	A	5.60	46	3	33	M	CXCR1/2
**49**	Male	48	A	5.90	51	3	7	n.d.	CXCR1/2
**50**	Female	57	A	9.30	61	4	23	n.d.	CXCR1/2
**51**	Female	52	A	7.10	78	5	7	M	CXCR1/2
**52**	Male	52	A	9.30	75	0.7	1	M	CXCR1/2
**53**	Male	65	A	7.90	59	38	7	n.d.	CXCR1/2
**54**	Male	80	A	86.0	94	83	84	n.d.	CXCR1/2
**55**	Female	54	A	12.0	80	1	2	n.d.	CXCR1/2
**56**	Female	69	B	5.30	50	0.5	47	n.d.	CXCR1/2

*Percentage of CD19^+^ (B cells, more than 99% CLL cells) in peripheral blood lymphocytes. ^Δ^Percentage of CD38^+^ cells in CD19^+^ lymphocytes. ^λ^Percentage of CD49d^+^ cells in CD19^+^ lymphocytes. ^I^Measured by flow cytometry; ^E^Measured by ELISA. IGHV immunoglobulin heavy chain variable region; M, mutated; U, unmutated. n.d., indicates not determined.

It is now clear that CLL cells located in different anatomic compartments express particular sets of genes^17–19^. Thus, leukemic B cells in lymphoid tissues have increased expression of B-cell activation genes compared to peripheral blood CLL cells due to the positive signals they received from the microenvironment. To address the possibility that activation of leukemic cells could induce the expression of IL-8 receptors, we incubated PBMC from CLL patients for 24 h in plates coated with anti-IgM antibodies to elicit extensive cross-linking of the B cell receptor. Soluble CD40 ligand (CD40L) was added to further increase activation signaling. As expected, this treatment resulted in the up-regulation of the activation marker CD69 in CLL cells (Fig. 1c and Supplementary Fig. S2), but it was unable to induce the expression of CXCR1 or CXCR2 (Fig. 1d). We conclude that resting or activated CLL cells do not express IL-8 receptors.

### IL-8 does not protect CLL cells from apoptosis *in vitro*

**Figure 2:**
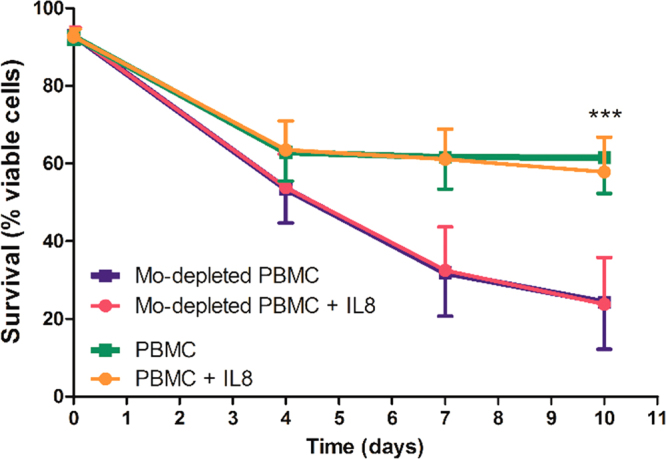
IL-8 does not prolong CLL cell survival *in vitro*. PBMC or monocyte-depleted PBMC (3 × 10^6^/ml) were incubated with or without recombinant IL-8 (20 ng/ml) for 10 days. IL-8 was added on days 0, 3, 6 and 9. Cell death of CD19^+^ lymphocytes was evaluated by flow cytometric alterations of light-scattering properties and confirmed by Annexin V staining on days 4, 7 and 10. Shown are mean ± SEM, n = 9. Statistical analysis was performed using Friedman test followed by the Dunn´s multiple comparison post-test. Asterisks indicate statistically significant differences (***p < 0.001) between PBMC and monocyte-depleted PBMC. The addition of IL-8 did not significantly modify cell survival of PBMC or monocyte-depleted PBMC.

Although we did not observe expression of CXCR1 or CXCR2 on leukemic B cells we could not rule out an alternative IL-8 binding site. Therefore we next determined if recombinant IL-8 was able to delay spontaneous CLL cell apoptosis. For these experiments, the concentration of IL-8 (20 ng/ml) was chosen based on previous data showing increased survival of CLL cells with doses ranged from 5 to 50 ng/ml^7^. In addition, we corroborated that this dose was effective because it was able to downregulate the expression of CXCR1 and CXCR2 on neutrophil membrane as a consequence of ligand-induced endocytosis^20^ (Supplementary Fig. S3). Given that monocytes differentiate *in vitro* into nurse-like cells that exert pro-survival effects on leukemic B cells^2^, experiments were performed not only with purified CLL cells, but also with PBMC. As expected, CLL cell viability was significantly higher in the presence of monocytes/nurse-like cells (Fig. 2). Moreover, and in agreement with previous results^7,21,22^, we observed variable rates of spontaneous cell death among CLL samples. Nevertheless, addition of exogenous IL-8 to cell cultures did not modify CLL cell survival under any of the experimental conditions tested. These results suggest that IL-8 does not exert a direct or indirect protective effect on circulating leukemic B cells.

### Leukemic B cells from CLL patients do not produce IL-8

**Figure 3:**
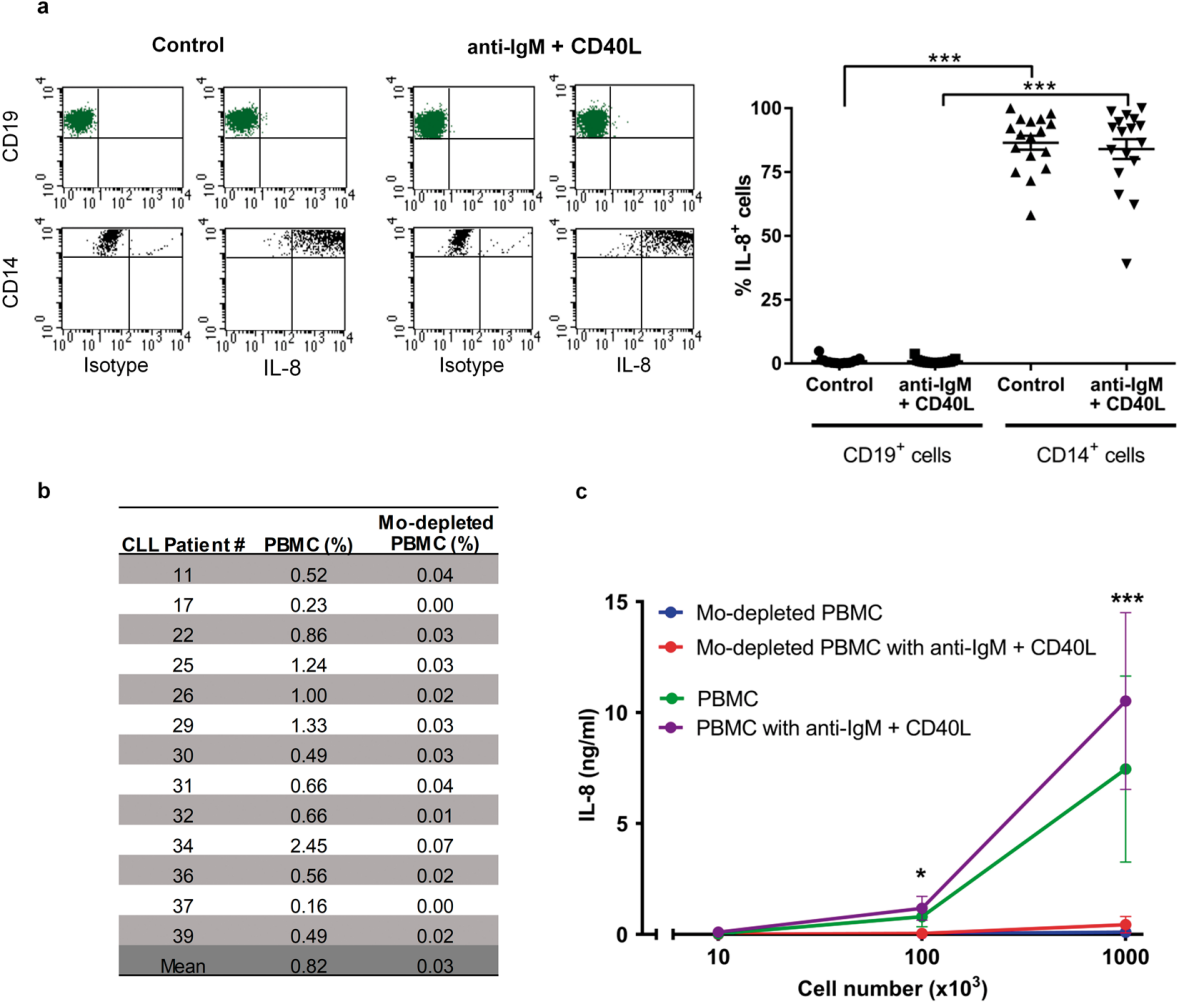
Monocytes, but not leukemic B cells, produce IL-8 in PBMC samples from CLL patients. (**a**) PBMC (3 × 10^6^/ml) were incubated with immobilized anti-IgM plus CD40L (40 ng/ml) or medium alone (control) for 24 h. Monensin (20 μM) was added for the last 4 h. to inhibit Golgi transport. IL-8 expression was evaluated by intracellular staining in CD19^+^ and CD14^+^ cells. Shown are representative dot plots from one CLL sample (left) and the percentages of IL-8 positive cells (mean ± SEM, n = 17) (right). (**b**) Aliquots of PBMC from 13 CLL samples were depleted from monocytes as described in Methods. The percentage of monocytes in each sample before and after depletion is shown. (**c**) Different cell numbers of PBMC or the corresponding monocyte-depleted PBMC were incubated with immobilized anti-IgM plus CD40L (40 ng/ml) or medium alone (control) for 24 h. The levels of IL-8 released to supernatants were quantified by ELISA. Results show the mean ± SEM of the 13 CLL samples. Statistical analysis was performed using Friedman test followed by the Dunn post-test. Asterisks indicate statistically significant differences between PBMC and monocyte-depleted PBMC (*p < 0.05; ***p < 0.001), both under control or activated conditions.

Next we determined if CLL cells are able to produce IL-8 spontaneously or upon stimulation. To this aim, PBMC from CLL patients were activated with anti-IgM plus CD40L as previously described. IL-8 production was evaluated by intracellular staining in CD19^+^ cells (CLL cells) and, as a positive control, in CD14^+^ cells (monocytes) within the same sample. These experiments were performed with PBMC from 17 CLL patients at different stages of the disease and belonging to low or high risk groups according to IGHV mutational status, CD38 and CD49d expression levels^1,2^ (Table 1). We found that, while CLL cells responded to stimulation by up-regulating the activation marker CD69, they were unable to produce IL-8, as only a very small proportion of CD19^+^ cells stained positive for IL-8 (% of CD19^+^IL-8^+^ cells in control versus activated conditions: 0.88 ± 0.27 vs 0.85 ± 0.21, mean ± SEM, n = 17). Representative dot plots of one CLL sample are depicted in Fig. 3a. To rule out the possibility that exposure to stimuli for 24 h was not long enough to induce IL-8 synthesis in CLL cells, we extended the cultures to 48 and 72 h with similar negative results (Supplementary Fig. S4). In addition, we used the prototypic TLR-2 ligand Pam3CSK4 (Pam3) to stimulate CLL cells since it was reported to induce IL-8 secretion in B cells from chronic inflammatory disease patients^23^. Once again we were unable to detect IL-8 production by CLL cells even though Pam3 significantly increased the expression of CD69 (Supplementary Fig. S5). As expected, we observed strong expression of IL-8 by monocytes, both in cultures exposed to anti-IgM plus CD40L or medium alone (Fig. 3a). Most likely, IL-8 synthesis in the absence of exogenous stimulus was due to activation of monocytes by adherence to plastic as previously reported^24^. Although the percentages of monocytes in the CLL samples evaluated were small (Fig. 3b), their robust capacity to produce IL-8 might explain the reported detection of the cytokine by ELISA^8,22,25,26^. To determine the actual contribution of monocytes to IL-8 levels detected in PBMC supernatants, we first removed monocytes from CLL samples and then we activated the leukemic cells as described above. The percentage of monocytes in PBMC samples before and after depletion is shown in Fig. 3b. We found high levels of IL-8 at 24 h in supernatants from PBMC incubated with or without anti-IgM plus CD40L (Fig. 3c). More importantly, IL-8 levels in supernatants decreased drastically after thorough depletion of monocytes (Fig. 3c), suggesting that they were the major (or even the unique) source of IL-8 in these cultures.

Altogether, our results demonstrate that CLL cells are not able to respond to IL-8 stimulation because they do not express the specific receptors CXCR1 and CXCR2, nor they seem to secrete IL-8 spontaneously or upon stimulation through their BCR or TLR-2. Although we cannot rule out that other stimuli from the microenvironment might induce the synthesis of IL-8 by CLL cells, the analysis of gene expression data obtained by Herishanu *et al*.^18^ (GEO #GSE21029) and Careta *et al*.^27^ (GEO #GSE30896) indicate that IL-8 and its receptors are not differentially expressed by leukemic B cells from lymphoid tissues compared to peripheral blood (Supplementary Fig. S6). On the other hand, due to the huge capacity of monocytes to release IL-8, they are most likely responsible for IL-8 production attributed to leukemic B cells. In fact, two independent studies have shown that higher levels of IL-8 in plasma from CLL patients do not correlate with lymphocytosis^5,28^. Whether increased concentration of IL-8 correlates with absolute numbers of blood monocytes warrant further investigation. Monocytes and macrophages are crucial for the pathogenesis of CLL^29–31^ and their increased numbers have been associated with inferior clinical outcomes by different research groups^30,32,33^. IL-8 secreted by monocytes and macrophages may affect CLL progression in many ways, including promotion of angiogenesis^34^ and attraction of myeloid cells to proliferation centers in lymphoid organs^29^. We have recently reported that increased levels of IL-8 in plasma of CLL patients prime neutrophils to release neutrophil extracellular traps (NETs) which delay spontaneous leukemic B cell apoptosis^6^. In conclusion, our findings do not diminish the relevance of IL-8 in CLL biology but suggest that its source and targets are different from the malignant clone.

## Methods

### Reagents and antibodies

RPMI 1640, fetal calf serum (FCS), penicillin and streptomycin were purchased from GIBCO. The Ficoll-Paque Plus used for cell separation was purchased from GE Healthcare (Munich, Germany). MACS CD14 cell isolation kit was obtained from Miltenyi Biotec. Bovine serum albumin was from Weiner Laboratorios (Santa Fe, Argentina). DMSO was purchased from Sigma-Aldrich (Dallas, USA). Annexin-V-FITC was obtained from ImmunoTools (Friesoythe, Germany). PE-conjugated mAbs anti- CD69 (clone FN50), anti-CD11b (clone D12), anti-CD49d (clone L25) and anti-CD38 (clone HB7), as well as FITC-anti-CD69 (clone FN50) and control Abs with irrelevant specificities (isotype control), monensin (BD GolgiStop™) and IL-8 ELISA Kit, were purchased from BD Biosciences (San Jose, USA). PC5-anti-CD19 (clone J3-119) was purchased from Beckman Coulter (Fullerton, USA). The following mAbs and recombinant human IL-8 were purchased from BioLegend (San Diego, USA): PE-anti-CD14 (clone HCD14), Alexa Fluor 488-anti-IL-8 (clone E8N1), FITC-anti-CXCR1 (clone 8F1) and PE-anti-CXCR2 (clone 5E8). Anti-human-IgM Ab was obtained from Jackson ImmunoResearch (West Grove, USA). Recombinant human CD40L was obtained from R&D Systems. Pam3CSK4 was purchased from Invivogen (San Diego, USA).

### CLL patients and healthy donor samples

Peripheral blood samples were collected from CLL patients and healthy donors after informed consent in accordance with the Declaration of Helsinki. These studies were approved by the Institutional Review Board of the National Academy of Medicine, Buenos Aires, Argentina. CLL was diagnosed according to standard clinical and laboratory criteria. At the time of analysis, all patients were free from clinically relevant infectious complications and were either untreated or had not received antineoplastic treatment for a period of at least six months.

### Cell separation procedures and culture

Peripheral blood leukocytes were isolated by centrifugation over a Ficoll–Paque layer. Mononuclear cells (PBMC) were recovered from the interface, washed twice with saline and suspended in complete medium (RPMI 1640 supplemented with 10% FCS and antibiotics). Cells were used immediately or were cryopreserved in FCS supplemented with 10% DMSO for further experiments. PBMC were depleted from monocytes (CD14^+^ cells) using the MACS CD14 cell isolation kit and the percentage of contaminating monocytes was checked using anti-CD11b mAb and flow cytometry analysis. Neutrophils were isolated by Ficoll-Paque centrifugation followed by dextran sedimentation. Contaminating erythrocytes were removed by hypotonic lysis. After washing with saline, cells (>96% viable neutrophils) were suspended in complete medium.

To induce CLL cell activation, PBMC or monocyte-depleted PBMC (3 × 10^6^ cells/ml) were incubated for 24 h on immobilized anti-human IgM (30 μg/ml) plus CD40L (40 ng/ml). Alternative Pam3CSK4 (100 ng/ml) was used to induce activation. As control, cells were cultured in complete medium. Activation was confirmed by evaluating expression of CD69 in CD19^+^ cells by flow cytometry.

### Analysis of CXCR1 and CXCR2 expression by flow cytometry

CXCR1 and CXCR2 expression on CLL cells were evaluated in freshly isolated PBMC or after thawing cryopreserved samples. In both cases, cell suspensions were incubated for 2 h at 37 °C before staining to allow potential re-expression of chemokine receptors. Immediately before staining, aliquots of neutrophils were added to PBMC suspensions as an internal positive control of CXCR1 and CXCR2. Analysis was performed using a FACScan flow cytometer (BD Immunocytometry Systems). Neutrophils and PBMC were discriminated by size (FSC-H) and internal complexity (SSC-H). Leukemic cells were further identified by CD19 expression.

### Evaluation of CLL cell survival *in vitro*

To evaluate the effect of IL-8 on cell survival, aliquots of 3 × 10^6^/ml PBMC or monocyte-depleted PBMC were cultured in complete medium for 10 days. Cultures were supplemented with human recombinant IL-8 (20 ng/ml) on days 0, 3, 6 and 9 of culture. Viability of CD19^+^ cells was evaluated by flow cytometric alterations of light-scattering properties and confirmed with Annexin V staining on days 0, 4, 7 and 10 of culture.

### Analysis of IL-8 production by flow cytometry and ELISA

For intracellular detection of IL-8 production, aliquots of 3 × 10^6^ PBMC were activated as described above and exposed to monensin (20 μM) for the last 4 h. Cells were then stained with anti-CD14 and anti-CD19 mAbs, were fixed with 1% of paraformaldehyde and permeabilized with 0.5% saponin in PBS. Finally cells were stained with anti-IL-8 or isotype-matched mAb and analyzed by flow cytometry. To ensure a proper analysis, at least 10,000 CD19^+^ cells and 3,000 CD14^+^ cells were collected separately. For quantification of IL-8 release, PBMC or monocyte-depleted PBMC (aliquots of 10^4^, 10^5^ or 10^6^ cells/well) were activated as described above with anti-IgM Ab plus CD40L for 24 h. IL-8 levels in supernatants were quantified by ELISA.

### Statistical analysis

Statistical significance was determined using the nonparametric tests: Wilcoxon signed rank test, Friedman test followed by the Dunn´s multiple comparison post-test and Mann-Whitney test. Data were analyzed using GraphPad Prism software version 6.01. In all cases, p < 0.05 was considered statistically significant.

### Data availability statement

All data generated or analyzed during this study are included in this published article (and its Supplementary Information files).

## Electronic supplementary material

Dataset1
