# Supplementary material for: Revisiting the role of interleukin-8 in chronic lymphocytic leukemia
Source: Sci Rep. 2017 Nov 16;7:15714. doi: 10.1038/s41598-017-15953-x (PMC5691131; doi:10.1038/s41598-017-15953-x)
Supplement: Supplementary file 1 — Dataset1 [file 41598_2017_15953_MOESM1_ESM.pdf]

Supplementary information

Revisiting the role of interleukin-8 in chronic lymphocytic leukemia

Denise Risnik[1], Enrique Podaza[1], María B. Almejún[1,2], Ana Colado[1], Esteban E. Elías[1],Raimundo F Bezares[3], Horacio Fernández-Grecco[4], Santiago Cranco[5], Julio C. Sánchez-Ávalos[5], Mercedes Borge[1,6], Romina Gamberale[1,6] and Mirta Giordano*[1,6]

[1]Laboratorio de Inmunología Oncológica, Instituto de Medicina Experimental (IMEX)-CONICET-Academia Nacional de Medicina, Buenos Aires, [2]Departamento de Fisiología, Biología Molecular y Celular, Facultad de Ciencias Exactas y Naturales, Universidad de Buenos Aires, [3]Hospital General de Agudos Dr. Teodoro Álvarez, Buenos Aires, [4]Sanatorio Municipal Dr. Julio Méndez, Buenos Aires, [5]Instituto Alexander Fleming, Buenos Aires, [6]Departamento de Microbiología, Parasitología e Inmunología, Facultad de Medicina, Universidad de Buenos Aires, Argentina.

Figure S1.

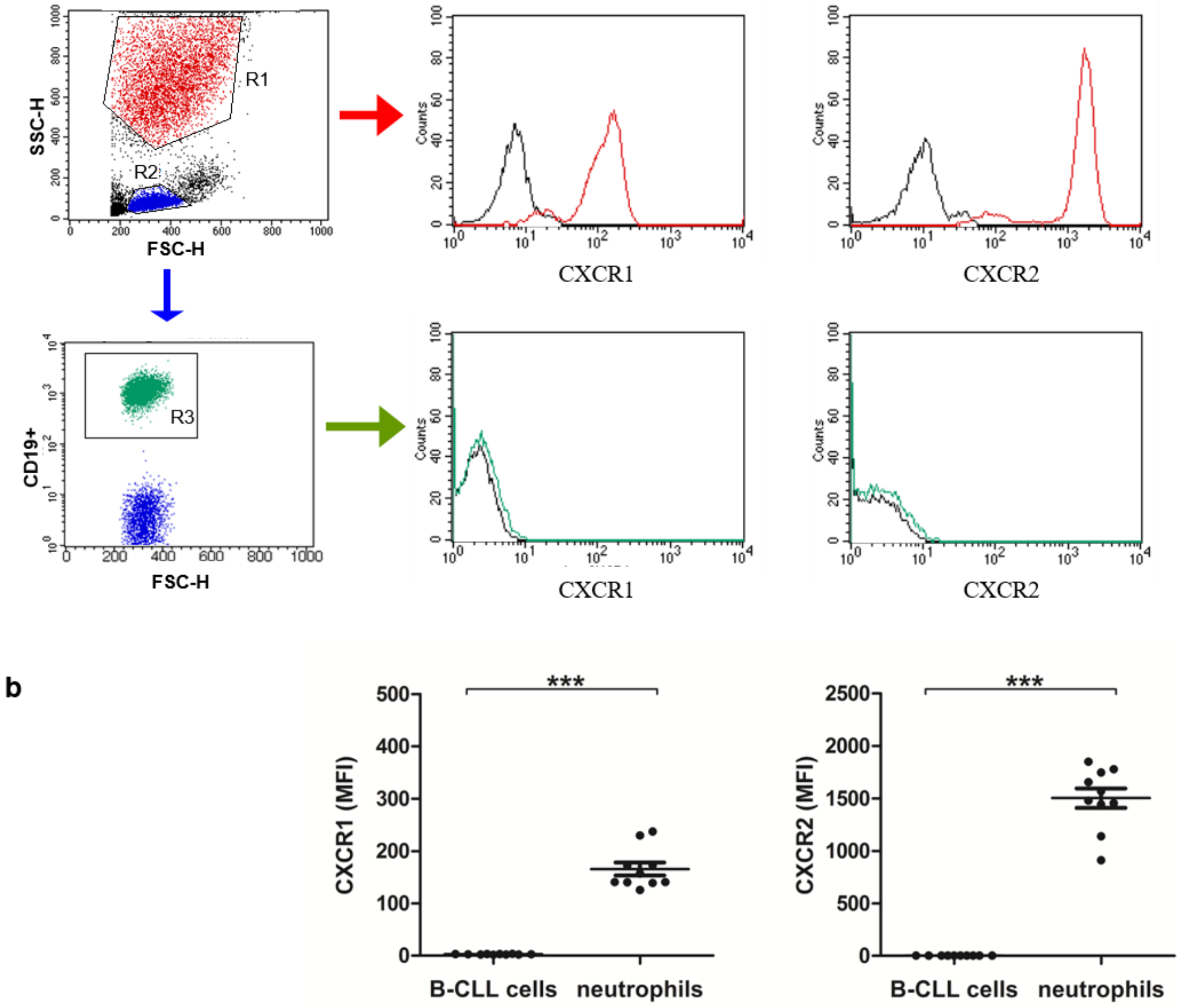

Supplementary Figure 1: CXCR1 and CXCR2 expression evaluated in whole blood from CLL patients.

Aliquots of whole blood samples from CLL patients were stained with anti-CD19 PC5, Anti-CXCR1 FITC and anti-CXCR2 PE and analyzed by flow cytometry. (a) Shown are dot-plots and histograms from one representative sample. Neutrophils (R1) and PBMC (R2) were first discriminated by size (FSC-H) and internal complexity (SSC-H). Leukemic cells were further identified by CD19 expression (R3). Isotype controls are depicted in grey. (b) Graphs show mean fluorescence intensity (MFI) of CXCR1 and CXCR2 in neutrophils and CLL cells (mean ± SEM, n=10). Statistical analysis was performed using Mann-Whitney test. Asterisks indicate statistically significant differences (*** $p<0.001$).

Figure S2.

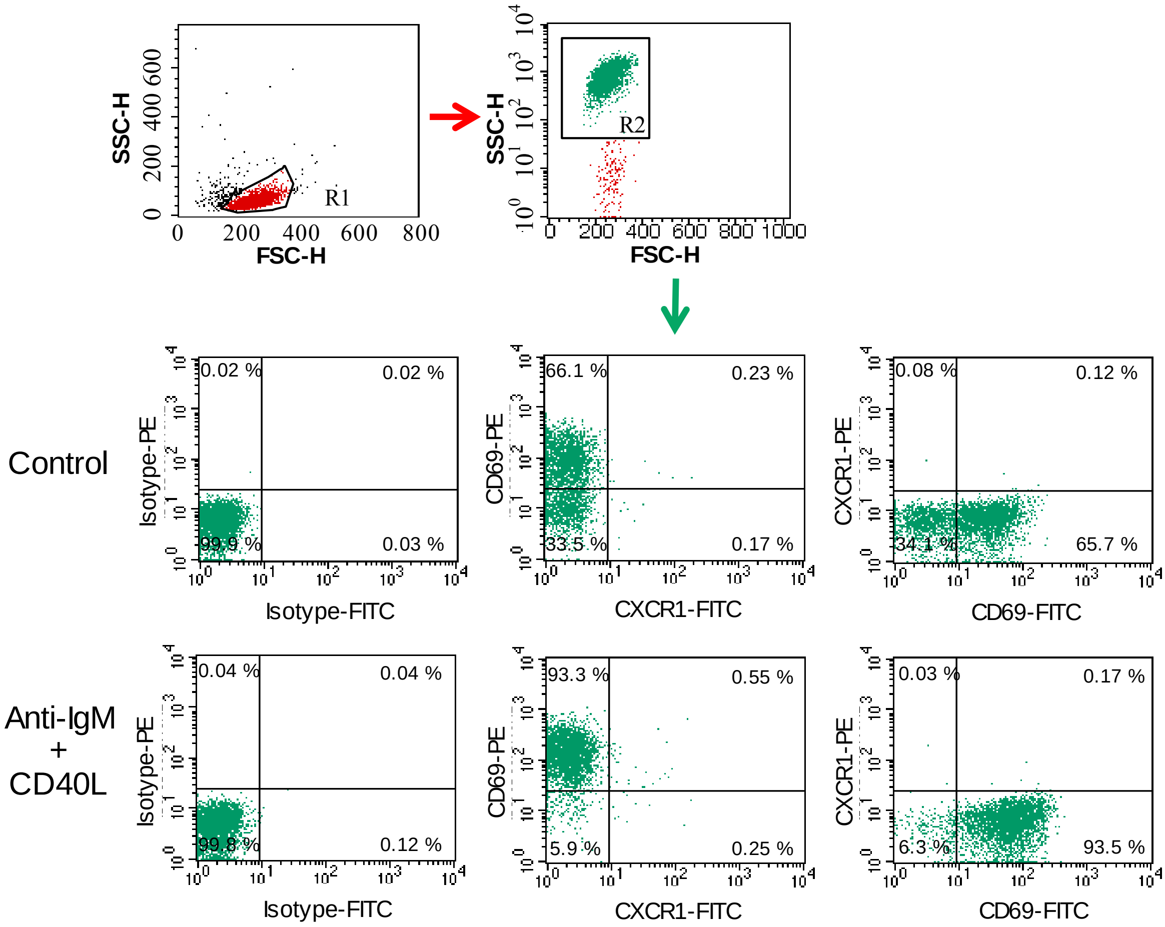

Supplementary Figure 2: CXCR1 and CXCR2 expression evaluated in activated B-CLL cells.

Patients PBMC were stimulated with anti-IgM and CD40L for 24 h. Unstimulated PBMC from the same patients were also culture in identical conditions (Control). Then cells were stained with anti-CD19 PC5, anti-CD69 PE and anti-CXCR1 FITC or anti-CD19 PC5, anti-CD69 FITC and anti-CXCR2 PE. Analysis was performed by flow cytometery. Lymphocytes (R1) were discriminated by size (FSC-H) and internal complexity (SSC-H). Leukemic cells (R2) were further identified by CD19 expression. Shown are representative dot-plots from one CLL sample (n=10).

Figure S3.

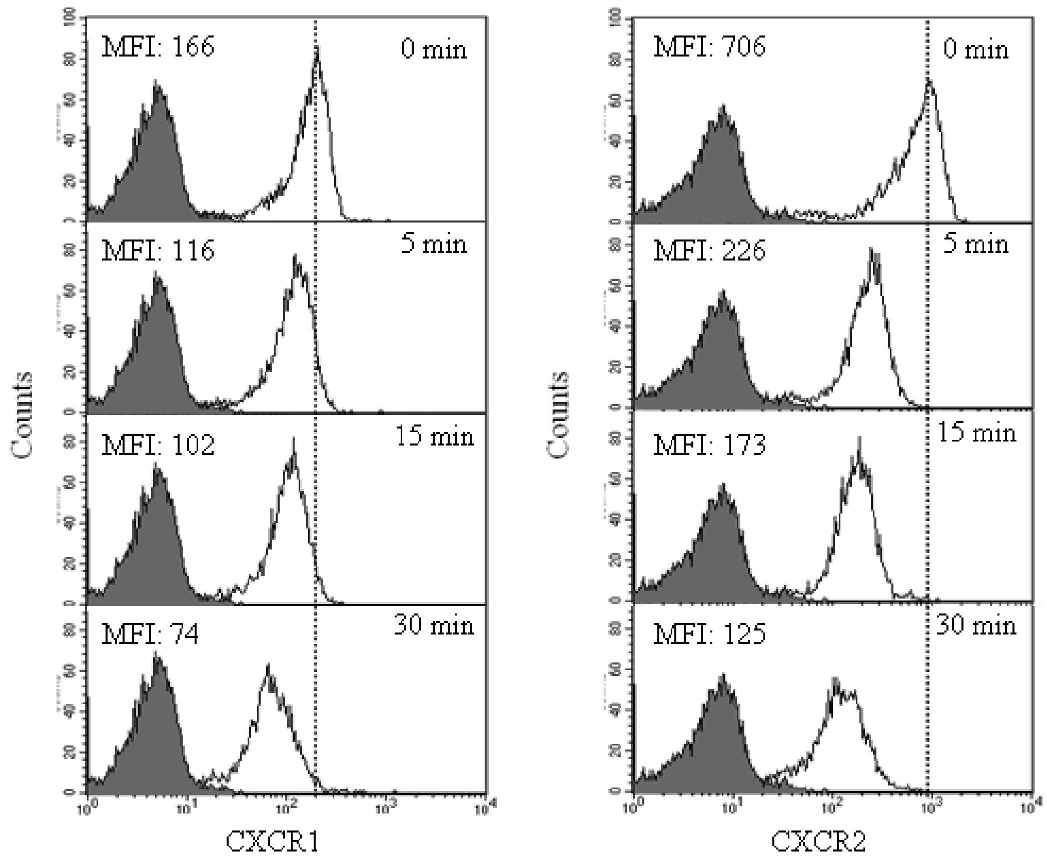

Supplementary Figure 3: Downregulation of CXCR1 and CXCR2 from neutrophil surface induced by IL-8.

Whole blood from healthy donor samples (n=3) was incubated with IL-8 (20 ng/ml) for 5, 15 or 30 min at 37ºC to induce the endocytosis of CXCR1 and CXCR2. After erythrocytes lysis, both IL-8 receptors were stained and analyzed by flow cytometry. Shown are representative histograms corresponding to neutrophil region discriminated by FSC-H and SSC-H parameters. Mean fluorescence intensity (MFI) for CXCR1 and CXCR2 at different time post IL-8 is indicated at the upper left corner. Isotype Ig control is depicted in grey.

Figure S4.

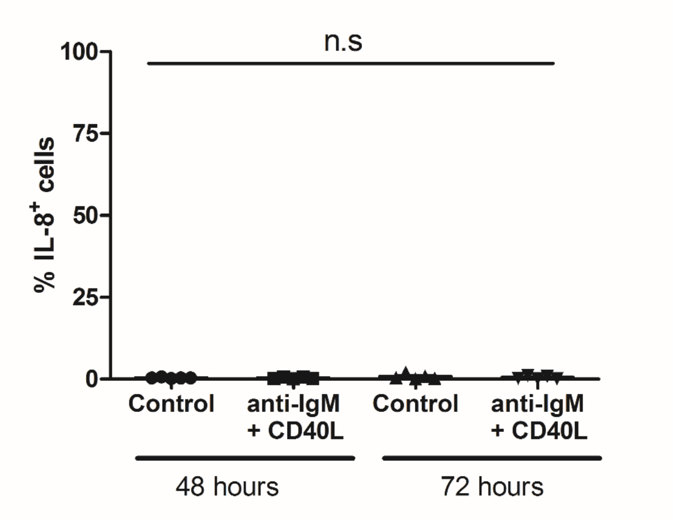

Supplementary Figure 4: CLL cells activated for 48-72 hours do not produce IL-8. PBMC ($3x10^6$/ml) from CLL samples (n=5) were incubated with immobilized anti-IgM plus CD40L (40 ng/ml) or medium alone (control) for 48-72 h. IL-8 production was evaluated by intracellular staining on $CD19^+$ cells. Shown are percentages of $IL\text{-}8^+$ $CD19^+$ cells. Statistical analysis was performed using Friedman test followed by the Dunn post-test.

Figure S5.

a

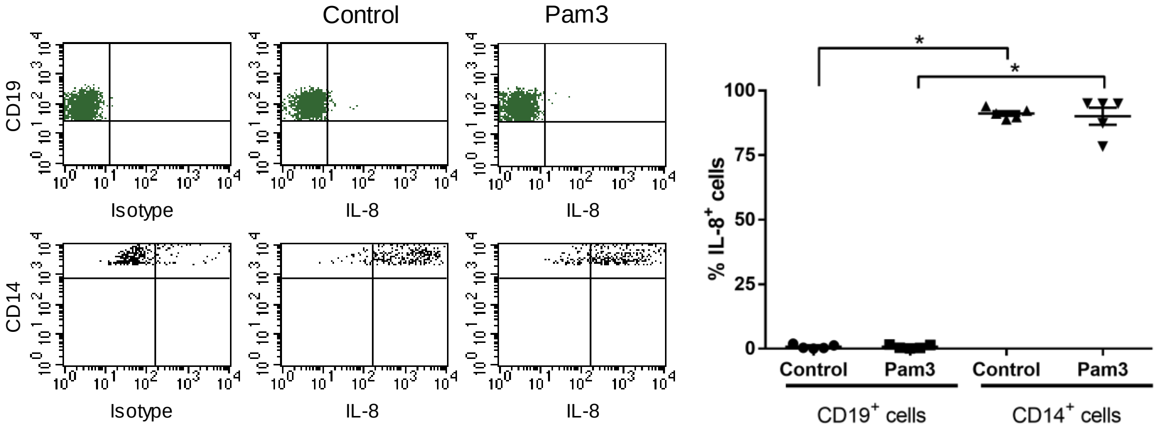

b

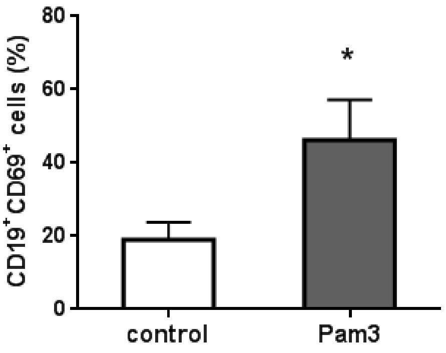

Supplementary Figure 5: Pam3 activates CLL cells without inducing IL-8 production.

PBMC ($3x10^6$/ml) from CLL samples were incubated with Pam3 (100 ng/ml) or medium alone (control) for 24 h. Monensin (20 µM) was added for the last 4 h. IL-8 expression was evaluated by intracellular staining in $CD19^+$ and $CD14^+$ cells. (a) Shown are representative dot plots from one CLL sample and the percentages of IL-8 positive cells (mean ± SEM, n=5). Statistical analysis was performed using Friedman test followed by the Dunn post-test (* $p<0.05$). (b) The expression of the activation marker CD69 on CD19+ cells was evaluated before adding monensin (mean ± SEM, n=4). Statistical analysis was performed using Wilcoxon signed-rank test (* $p<0.05$).

Figure S6.

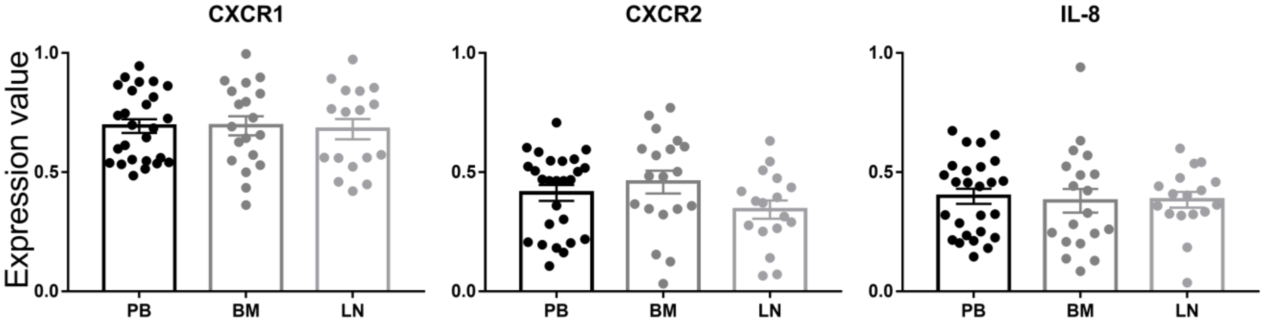

Supplementary Figure 6: IL-8, CXCR1 and CXCR2 are not differentially expressed in CLL cells from peripheral blood (PB), bone marrow (BM) and lymph nodes (LN) samples.

Comparison of gene expression from the #GSE21029 database was performed in the GEO platform. Shown are expression values from individual samples and mean ± SEM, n=17-26. Statistical analysis was performed using Friedman test followed by the Dunn post-test ($p>0.05$).
